# Adverse Childhood Experiences, Commitment Offense, and Race/Ethnicity: Are the Effects Crime-, Race-, and Ethnicity-Specific?

**DOI:** 10.3390/ijerph14030331

**Published:** 2017-03-22

**Authors:** Matt DeLisi, Justin Alcala, Abdi Kusow, Andy Hochstetler, Mark H. Heirigs, Jonathan W. Caudill, Chad R. Trulson, Michael T. Baglivio

**Affiliations:** 1 Department of Sociology, Iowa State University, Ames, IA 50011, USA; jd0g93@iastate.edu (J.A.); kusow@iastate.edu (A.K.); hochstet@iastate.edu (A.H.); mheirigs@iastate.edu (M.H.H.); 2 Department of Public Affairs, University of Colorado, Colorado Springs, CO 80918, USA; jcaudill@uccs.edu; 3 Department of Criminal Justice, University of North Texas, 1155 Union Cir, Denton, TX 76203, USA; chad.trulson@unt.edu; 4 G4S Youth Services, LLC, Tampa, FL 33634, USA; michael.baglivio@us.g4s.com

**Keywords:** adverse childhood experiences, delinquency, crime, race, ethnicity, juvenile justice

## Abstract

Adverse childhood experiences are associated with an array of health, psychiatric, and behavioral problems including antisocial behavior. Criminologists have recently utilized adverse childhood experiences as an organizing research framework and shown that adverse childhood experiences are associated with delinquency, violence, and more chronic/severe criminal careers. However, much less is known about adverse childhood experiences vis-à-vis specific forms of crime and whether the effects vary across race and ethnicity. Using a sample of 2520 male confined juvenile delinquents, the current study used epidemiological tables of odds (both unadjusted and adjusted for onset, total adjudications, and total out of home placements) to evaluate the significance of the number of adverse childhood experiences on commitment for homicide, sexual assault, and serious persons/property offending. The effects of adverse childhood experiences vary considerably across racial and ethnic groups and across offense types. Adverse childhood experiences are strongly and positively associated with sexual offending, but negatively associated with homicide and serious person/property offending. Differential effects of adverse childhood experiences were also seen among African Americans, Hispanics, and whites. Suggestions for future research to clarify the mechanisms by which adverse childhood experiences manifest in specific forms of criminal behavior are offered.

## 1. Introduction

In the seminal survey of 9508 adults in the Adverse Childhood Experiences (ACE) study, Felitti and colleagues [1] introduced the concept of adverse childhood experiences to account for the negative health and behavioral consequences of various forms of childhood abuse, neglect, and exposure to household dysfunction. The seminal ACE study and subsequent studies [2,3,4,5] supported the notion that individuals who endured more adverse childhood experiences tended to suffer the most throughout the life course and evinced the greatest number of health problems, maladaptive behaviors, and comorbid psychiatric conditions.

One of the maladaptive behaviors that results from adverse childhood experiences is antisocial behavior. In recent years, criminologists have utilized the adverse childhood experiences conceptual framework to examine associations with delinquency, crime, and serious violence. It has become clear that diverse adverse childhood experiences are fairly pervasive among clinical and correctional samples relative to those in the general population. For example, Baglivio and colleagues [6] examined a sample of more than 60,000 juvenile offenders from the United States and found that delinquents were significantly more likely than those in the general population to have pervasive adverse childhood experiences and significantly less likely to never experience adverse childhood experiences. In other words, clinical samples of youth evince a high preponderance of abusive experiences and deprivation. These findings were consistent with studies of youth residing in detention, correctional, and confinement facilities [7,8,9,10,11] where adverse childhood experiences are endemic.

There is also compelling evidence that adverse childhood experiences are particularly serious risk factors for more pathological forms of offending. For instance, Fox and colleagues [12] reported that each additional adverse childhood experience increased the likelihood of serious, violent, and chronic juvenile offending by 35%. Others have similarly found that adverse childhood experiences were linked to more severe offending trajectories, earlier onset of antisocial conduct, and shorter time to recidivism post-juvenile justice services [13,14]. For instance, Boduszek and colleagues [15] found that male prisoners in Poland who had been exposed to family violence were approximately six times more likely to perpetrate a homicide than offenders who lacked violence exposure. In terms of sexual violence, a host of investigators have found that assorted adverse childhood experiences, particularly childhood sexual abuse victimization, were associated with an increased likelihood of perpetrating sexual crimes during adolescence or adulthood [16,17,18,19,20,21]. The evidence is clear that adverse childhood experiences increase the liability for externalizing symptoms and diverse forms of antisocial conduct.

Despite these general findings, there remain important knowledge gaps. For instance, in the United States there is extraordinary heterogeneity of background experiences among delinquents in addition to sharply divergent local life circumstances and socioeconomic backgrounds by race and ethnicity [22,23,24,25,26], which have been demonstrated to affect adverse childhood experience wherein juvenile offenders residing in more disadvantaged communities evidence greater exposures. Prior criminological research has shown that disaggregated analyses by race and ethnicity are a fruitful way to understand different pathways of offending [27,28,29]. Given these varying background experiences, it is unclear to what extent adverse childhood experiences are associated with crime in the same way for different racial and ethnic groups, and to what degree the adverse childhood experience effects are potentially crime-specific. For example, Duke and colleagues [30] examined 136,549 youth who responded to the 2007 Minnesota Student Survey and found differential associations between adverse childhood experiences by race and gender in addition to differential effects on specific forms of antisocial behavior, including delinquency, bullying, physical fighting, dating violence, and weapons carrying. Adverse childhood experiences were negatively associated with delinquency, but positively associated with various forms of violence and weapons carrying. To our knowledge, no prior study has examined the adverse childhood experiences-crime link using models that are disaggregated by race, ethnicity, and offense type. The current study was conducted to examine three research questions: (1) whether there are differential effects for the number of adverse childhood experiences that a youth experienced; (2) whether these effects vary across race and ethnic groups, and (3) whether the adverse childhood experiences-antisocial behavior association depends on the commitment offense type.

## 2. Materials and Methods

### 2.1. Participants and Procedures

Cross-sectional data are based on a sample of (*n* = 2520) adjudicated male delinquents committed to confinement facilities in a large southern state in 2009. Information on each juvenile offender was compiled by the juvenile correctional system at a statewide intake unit upon the youths’ commitment and during their institutionalization. All state-committed youth were housed at the intake facility for approximately two months and then were transferred to specific facilities around the state to complete their commitment period. Additional offender data were collected during the youth’s confinement from numerous sources including state-level and county-level official records, on-site diagnostic procedures at intake, observations from professional and correctional staff, self-report information from youth, or a combination of these sources. Delinquency history and additional file information for all state-committed delinquents were collected with standardized instruments at each juvenile facility in the state and maintained at a centralized location.

### 2.2. Measures

#### 2.2.1. Adverse Childhood Experiences

A count-measure of adverse childhood experience exposures (Mean = 2.08, Standard Deviation = 1.51, range = 0–7) was constructed from seven dichotomous variables indicating (1) whether the youth had been physically abused; (2) whether the youth had been sexually abused; (3) whether the youth had been emotionally abused; (4) whether the youth was reared in poverty; (5) whether the youth was reared in a chaotic home characterized by residential instability and multiple family members and acquaintances living in the home; (6) whether the youth had family members who were in gangs; and (7) whether the youth had been violent toward members of his immediate family. The use of multiple and diverse indicators of adverse childhood experiences is consistent with prior research [31,32]. In terms of prevalence of adverse childhood experiences, 17.3% had zero, 18% had one, 30.9% had two, 17.1% had three, 8.7% had four, 5.8% had five, 1.82% had six, and 0.3% had all seven.

#### 2.2.2. Race and Ethnicity

Analyses were performed separately for African Americans (*n* = 889, 35.3%), Hispanics (*n* = 966, 38.3%), and whites (*n* = 625, 24.8%).

#### 2.2.3. Commitment Offense Type

Commitment offense type is the most serious charge for which the ward was committed to the confinement facility. Homicide offenses (*n* = 681, 27.02%) included capital murder, attempted capital murder, murder, attempted murder, criminally negligent homicide, and voluntary manslaughter. Sexual offenses (*n* = 930, 36.9%) included aggravated sexual assault, attempted aggravated sexual assault, attempted sexual assault, and sexual assault). Serious person/property offenses (*n* = 498, 19.76%) included aggravated robbery and attempted aggravated robbery.

#### 2.2.4. Confounders

Three delinquent career parameters were used as statistical controls in the adjusted tables of odds based on their empirical associations with serious delinquency [33,34,35,36,37]. These were age at first commitment to a confinement facility which captures onset (Mean = 15.29, Standard Deviation = 1.14, range = 10–18), total previous delinquent adjudications (Mean = 1.60, Standard Deviation = 0.95, range = 0–10), and total prior out of home placements (Mean = 0.45, Standard Deviation = 1.12, range = 0–15).

### 2.3. Analytical Technique

Epidemiological tables of odds were used to examine the associations between adverse childhood experiences and the three commitment offense types for the full sample, African Americans, Hispanics, and whites. Epidemiological tables of odds are used for case-control and cross-sectional data to evaluate the odds of a binary outcome (e.g., commitment for homicide, sexual, or serious person/property offense) by score on a predictor variable (e.g., adverse childhood experiences). Two additional tests were conducted. The test of homogeneity evaluates if the odds of failure associated with adverse childhood experiences the youth experienced are equal. The score test for trend of odds indicates the positive or negative trend in the association between adverse childhood experiences and the commitment offense types.

Two sets of models were performed. First, an unadjusted model showed the associations of adverse childhood experiences on commitment offense type. Second, adjusted models controlling for the three delinquent career confounders were used with Mantel-Haenszel odds ratios.

## 3. Results

### 3.1. Homicide

As shown in Table 1, adverse childhood experiences were variously associated with homicide as a commitment offense. For the total sample in the unadjusted model, wards with five adverse childhood experiences were 49% less likely and wards with six adverse childhood experiences were 72% less likely to be committed for homicide. Indeed, none of the adverse childhood experiences had an odds ratio >1 for the total sample. The test of homogeneity was significant, indicating there were not equal odds across scores on adverse childhood experiences, and the score test for the trend of odds was significant and revealed a negative association between adverse childhood experiences and homicide. In the adjusted model, the only significant association was for wards with six adverse childhood experiences who were 68% less likely to be committed for homicide.

The effects of adverse childhood experiences were inconsistently related to homicide across racial and ethnic groups. In the unadjusted model, African Americans with one adverse childhood experience were 39% less likely to be committed for homicide. In the adjusted model, Hispanics with four adverse childhood experiences were two times more likely to be committed for homicide. In the unadjusted model, whites with five (68% less likely) and six (85% less likely) adverse childhood experiences evinced a reduced likelihood of homicide offending.

### 3.2. Sexual Offense

As shown in Table 2, there were dramatic associations between adverse childhood experiences and being committed for a sexual offense; however, the effects varied across models. For the total sample in both the unadjusted and adjusted models, wards that had two or more adverse childhood experiences were more likely to be committed for a sexual offense. Indeed, those with seven adverse childhood experiences had an odds ratio of 6.3 for sexual offending. The adjusted models showed disparate effects. African Americans with three (Odds Ratio = 1.76) and five (Odds Ratio = 4.67) adverse childhood experiences were more likely to be committed for a sexual offense. Hispanics with two to six adverse childhood experiences were more likely to be committed for a sexual offense and the trend was positive. For whites, only those with five adverse childhood experiences were significantly likely to be committed for a sexual offense and the trend was positive and linear.

### 3.3. Serious Person/Property Offending

As shown in Table 3, adverse childhood experiences mostly showed a negative association with serious person/property offending. For the total sample in both the unadjusted and adjusted models, wards with three, four, five, and six adverse childhood experiences were negatively associated with serious person/property offending. For instance, those with three adverse childhood experiences were 44% less likely to be committed for serious person/property offending. The likelihood was 49% less likely for four adverse childhood experiences, 60% less likely for five adverse childhood experiences, and 84% less likely for six adverse childhood experiences. No significant effects were found in the adjusted model for African Americans. However, Hispanics with three adverse childhood experiences were 60% less likely to be committed for serious person/property offending, those with four experiences were 73% less likely, and those with five experiences were 78% less likely. For whites, different sorts of effects were found. Those with one adverse childhood experience were 387% more likely and those with two adverse childhood experiences were 329% more likely to be committed for serious person/property offending.

## 4. Discussion

Adverse childhood experiences have been shown to have long-term effects on behavioral functioning and have recently been utilized by criminologists as a conceptual framework to understand antisocial development [6,12,13,14,15,16,17,18,19,20]. Using a large sample of 2520 serious male juvenile delinquents, the current study examined whether there were differential effects on crime based on the number of adverse childhood experiences that a ward experienced, whether these effects varied across race and ethnic groups, and whether the adverse childhood experiences’ association with offending depended on the commitment offense type. The results revealed that adverse childhood experiences do not operate on criminal outcomes in a directly linear manner and have considerably differential effects.

First, it is not the case that a youth who has been exposed to the greatest number of adverse childhood experiences will necessarily be at the greatest risk of offending. Youth who had six adverse childhood experiences were 72% less likely to be committed for homicide, 413% more likely to be committed for a sexual offense, and 50% less likely to be committed for a serious person/property offense. Across models, these inconsistent effects were common. In the unadjusted model, African Americans with one adverse childhood experience were 39% less likely to be committed for homicide, whereas in the adjusted model for whites, those who had six adverse childhood experiences were 85% less likely to be committed for homicide. Still, there was also evidence of a gradient effect for crimes other than homicide. There was a clear positive trend where more adverse childhood experiences were associated with a greater likelihood of sexual offending, and in the total sample and among Hispanics, a negative trend where more adverse childhood experiences were associated with less risk for serious person/property offending.

Second, adverse childhood experiences had differential associations with the three offenses by racial and ethnic group. African Americans with one adverse childhood experience were 39% less likely to be committed for homicide in the unadjusted model. Hispanics with four adverse childhood experiences were 100% more likely to be committed for homicide. Whites with five or six adverse childhood experiences were 68% and 85% less likely, respectively, to be committed for homicide in the unadjusted model. There was more consistency in the effects for sexual offense where adverse childhood experiences were associated with elevated risk for sexual offending across racial and ethnic groups. However, even within this consistency, there were differences. Among wards with five adverse childhood experiences, the likelihood of being committed for sexual offense was 367% for African Americans, 314% for Hispanics, and 257% for whites. Whites with fewer adverse childhood experiences had extremely high likelihood of commitment of a serious person/property offense (Odds Ratio = 4.87 for one adverse childhood experience and Odds Ratio = 4.29 for two adverse childhood experiences), yet African Americans and Hispanics with between three and five adverse childhood experiences had reduced likelihood of serious person/property offending. Although non-white offenders experience generally more disadvantaged backgrounds [24,25,26,38] than whites, that was not the case in these current data. Whites had the highest mean adverse childhood experiences (Mean = 2.43, Standard Deviation = 1.71), followed by Hispanics (Mean = 2.01, Standard Deviation = 1.42) and African Americans (Mean = 1.94, Standard Deviation = 1.41), and these group differences were significant (*F*_(3, 2520)_ = 17.00, *p* < 0.0001). This finding is consistent with prior work which indicated a higher proportion of white juvenile offenders at the extreme upper end of the range of adverse childhood experiences (22% compared to 15% of African American youth and 11% of Hispanic youth) [22].

Third, adverse childhood experiences were not associated with homicide, sexual offending, and serious person/property offending in the same ways. The effects were mostly negative for homicide, were sharply positive for sexual offending, and were negative for serious person/property offending, with the exception of whites where the effects were positive. Other criminologists have also recently found evidence of adverse childhood experiences on specific forms of crime and negative behaviors. For instance, Perez and colleagues [39,40] found that adverse childhood experiences were directly associated with substance abuse and with serious, violent, and chronic delinquency in their study of more than 64,000 delinquent youth in Florida. However, the effects were rather small, with adverse childhood experiences increasing the likelihood of substance abuse by 14% and of serious, violent, and chronic delinquency by 8%. Additional research is needed to examine how and to what degree adverse childhood experiences are associated with variance in specific forms of serious crime, including homicide and aggravated robbery. Moreover, while there is considerable evidence that sexual abuse victimization can be a distal predictor of subsequent sexual offending [17,18,19,20,21], much less is known about how poverty, physical abuse, emotional abuse, and other deprivations are linked to other forms of violence.

There are clear policy applications from our findings. Any program that attempts to reduce various forms of childhood abuse and neglect, and to generally reduce the incidence of adverse childhood experiences is certainly a worthy endeavor. Even one type of adverse childhood experience can be the driving force in propelling a youth toward delinquent and other antisocial conduct. Central among these programs would be parenting education and training that provides adaptive, instructive ways for parents to interact with and discipline their children. These healthy alternatives engender a cascade of healthy development compared to the pernicious and enduring effects of abuse and neglect.

There are key limitations to the present study that should be considered to not only contextualize the findings, but also to guide future research. There are numerous important criminological variables, such as religiosity, family support, and various social bonds that we lacked measures of and thus were not able to specify them in the multivariate models. Although the adjusted models controlled for three important parameters of the delinquent career, there were omitted variables that would be helpful to specify the mechanisms by which adverse childhood experiences translate into elevated or attenuated risks for various types of serious delinquency. It is likely that the inconsistent ways that adverse childhood experiences manifest in crime in the present study are reflective of the mechanism advanced by differential susceptibility theory [41,42]. Some youth suffer considerably from even one adverse childhood experience, such as the case of whites in the current data and their risk for serious person/property offending, whereas others are able to avoid specific forms of crime despite many adverse childhood experiences, such as the case of whites in the current data and their risk for homicide. In their landmark study, Caspi and colleagues [43] revealed how specific genetic polymorphisms moderate abuse experiences and explain differential antisocial outcomes. Biosocial scholars should model adverse childhood experiences relative to well-known genetic risks for antisocial traits and behaviors, such as dopaminergic and serotonergic genes, to more clearly articulate for whom adverse childhood experiences are most damaging.

Another fruitful research endeavor is to examine adverse childhood experiences vis-à-vis psychopathic traits and serious offending. Anda and colleagues [44] found that persons with a score of five or more (maximum of eight) adverse childhood experiences had nearly a three-fold increase in psychopathic personality features. Given the strong association between psychopathy and the most severe forms of juvenile delinquency [45,46,47,48] and the association between psychopathy and instrumental forms of offending, such as murder, rape, and armed robbery, it is likely that some of the youth in the current study exhibited these traits, but we were unable to measure them. Moreover, a bevy of studies have revealed that psychopathic offenders often experience severely abusive and neglectful childhoods [49,50,51,52,53,54], which contributes to their pernicious antisocial development. However, no studies to our knowledge have explicitly examined the interplay between psychopathy and adverse childhood experiences relative to crime.

The current study examined only three types of offending; thus, future research should extend this by examining other types of offenses. There could be interesting developmental sequela associated with specific forms of offending. For instance, Dube and colleagues’ [55] study of the original ACE data found that each additional adverse childhood experience increased the likelihood of early initiation of drug use two- to four-fold, and those with the greatest number of adverse childhood experiences were seven to 10 times more likely to be early-onset drug users. However, it is likely that some adverse childhood experiences, such as parents who openly use drugs in front of their children or furnish drugs to their children, would produce a stronger association with drug crimes than poverty or emotional abuse, for instance. In addition, the coding of adverse childhood experiences might obscure variation. Counts used here reflect the diversity of experiences but not the frequency or severity of those experiences, nor the relationship of the youth to the perpetrator. It is possible that frequency and severity vary by subgroup and the resultant error from this omission contributes to the discrepant findings. Additional study of adverse childhood experiences and specific forms of crime could also inform the understanding of criminal specialization to the degree that a specific form of abuse is associated with subsequent offending, as in the case of sexual abuse/sexual assault [17,18,19,20].

## 5. Conclusions

Adverse childhood experiences are variously associated with commitment offenses and work in similar and dissimilar ways across racial and ethnic groups. Youth with more adverse childhood experiences were generally less likely to be committed for homicide or serious person/property offending but more likely to be committed for sexual offenses, particularly when models were adjusted for onset of first commitment, previous delinquent adjudications, and prior out of home placements. The effects of adverse childhood experiences on sexual offending are robust and work similarly for African Americans, Hispanics, and whites; however, there remained differential effect sizes. The study of adverse childhood experiences is an invaluable framework for criminology, and one where greater specificity of the mechanisms and ultimate effects of these experiences is needed.

## Figures and Tables

**Table 1:** Table of odds for homicide as commitment offense. ACE, Adverse Childhood Experiences. OR, Odds Ratio.

Adverse Childhood Experiences	Total Sample		African Americans		Hispanics		Whites	
ACE	Unadjusted OR (χ^2^)	Adjusted OR (χ^2^)	Unadjusted OR (χ^2^)	Adjusted OR (χ^2^)	Unadjusted OR (χ^2^)	Adjusted OR (χ^2^)	Unadjusted OR (χ^2^)	Adjusted OR (χ^2^)
0	Reference	Reference	Reference	Reference	Reference	Reference	Reference	Reference
1	0.86 (1.06)	0.94 (0.15)	0.61 (4.05) *	0.67 (2.30)	1.18 (0.50)	1.26 (0.81)	0.76 (0.60)	0.85 (0.15)
2	0.90 (0.61)	1.09 (0.39)	0.90 (0.28)	1.16 (0.46)	0.97 (0.03)	1.24 (0.81)	0.55 (2.91)	0.68 (0.82)
3	0.77 (2.91)	0.81 (1.45)	0.79 (1.03)	0.78 (0.78)	0.98 (0.01)	0.84 (0.28)	0.53 (3.01)	0.92 (0.04)
4	0.80 (1.52)	0.95 (0.07)	0.53 (3.36)	0.70 (0.83)	1.33 (0.99)	2.00 (4.27) *	0.63 (1.28)	0.39 (1.96)
5	0.51 (8.31) **	0.62 (3.57)	0.67 (1.10)	0.47 (3.02)	0.68 (0.96)	1.03 (0.00)	0.32 (5.12) *	0.52 (1.04)
6	0.28 (7.94) **	0.32 (4.19) *	0.58 (0.42)	0.73 (0.09)	0.45 (1.08)	0.88 (0.02)	0.15 (4.09) *	0 (2.70)
7	0.38 (0.87)	0.45 (0.72)	-	-	-	-	-	-
Test of Homogeneity	16.78 **	-	8.10	-	5.06	-	9.82	-
Score Test for Trend of Odds	12.24 ***	4.74 *	2.10	1.21	0.43	0.03	7.94 **	5.47 *

* *p* < 0.05; ** *p* < 0.01; *** *p* < 0.001.

**Table 2:** Table of odds for sexual offense as commitment offense.

Adverse Childhood Experiences	Total Sample		African Americans		Hispanics		Whites	
ACE	Unadjusted OR (χ^2^)	Adjusted OR (χ^2^)	Unadjusted OR (χ^2^)	Adjusted OR (χ^2^)	Unadjusted OR (χ^2^)	Adjusted OR (χ^2^)	Unadjusted OR (χ^2^)	Adjusted OR (χ^2^)
0	Reference	Reference	Reference	Reference	Reference	Reference	Reference	Reference
1	1.16 (0.96)	1.11 (0.41)	1.29 (0.92)	1.23 (0.49)	1.37 (1.37)	1.47 (1.46)	0.84 (0.41)	0.79 (0.50)
2	1.41 (6.89) **	1.32 (3.88) *	1.47 (2.79)	1.18 (0.47)	1.83 (6.36) **	1.95 (6.38) **	1.46 (2.01)	1.26 (0.57)
3	2.13 (27.41) ***	1.93 (17.93) ***	1.92 (6.60) **	1.76 (4.51) *	2.67 (13.85) ***	3.42 (14.46) ***	1.75 (3.97) *	1.57 (1.98)
4	2.34 (24.57) ***	1.92 (10.46) ***	1.90 (3.76) *	1.64 (1.42)	2.85 (11.99) ***	2.32 (4.91) *	1.67 (2.62)	2.35 (3.50)
5	4.20 (54.85) ***	4.50 (40.38) ***	2.81 (8.05) **	4.67 (11.59) ***	4.75 (19.44) ***	4.14 (12.18) ***	3.40 (11.36) ***	3.57 (7.56) **
6	5.13 (28.99) ***	3.65 (8.92) **	1.27 (0.08)	1.87 (0.38)	4.54 (6.96) **	5.69 (4.73) *	6.49 (10.06) ***	3.93 (2.30)
7	3.65 (3.20)	6.33 (3.73) *	-	-	-	-	-	-
Test of Homogeneity	100.97 ***	-	12.69	-	34.85 ***	-	28.08 ***	-
Score Test for Trend of Odds	94.16 ***	65.62 ***	10.81 ***	7.55 **	33.78 ***	29.63 ***	22.65 ***	16.01 ***

* *p* < 0.05; ** *p* <0.01; *** *p* < 0.001.

**Table 3:** Table of odds for serious person/property offense as commitment offense.

Adverse Childhood Experiences	Total Sample		African Americans		Hispanics		Whites	
ACE	Unadjusted OR (χ^2^)	Adjusted OR (χ^2^)	Unadjusted OR (χ^2^)	Adjusted OR (χ^2^)	Unadjusted OR (χ^2^)	Adjusted OR (χ^2^)	Unadjusted OR (χ^2^)	Adjusted OR (χ^2^)
0	Reference	Reference	Reference	Reference	Reference	Reference	Reference	Reference
1	1.31 (2.92)	1.29 (2.44)	1.09 (0.14)	0.98 (0.00)	1.05 (0.04)	1.00 (0.00)	5.88 (11.84) ***	4.87 (9.12) **
2	1.05 (0.13)	0.98 (0.02)	0.94 (0.08)	0.90 (0.21)	0.79 (1.08)	0.64 (3.07)	3.75 (6.09) **	4.29 (6.69) **
3	0.54 (11.64) ***	0.56 (7.90) **	0.52 (6.00) **	0.57 (3.22)	0.42 (8.09) **	0.40 (6.09) **	1.69 (0.71)	2.19 (1.11)
4	0.51 (8.52) **	0.51 (6.14) **	0.65 (1.44)	0.56 (1.72)	0.28 (9.08) **	0.27 (7.56) **	2.42 (1.96)	1.28 (0.08)
5	0.50 (6.34) **	0.40 (7.27) **	0.50 (2.43)	0.53 (1.36)	0.40 (3.27)	0.22 (4.92) **	2.47 (1.94)	2.30 (0.92)
6	0.34 (4.34) *	0.16 (4.47) *	1.42 (0.22)	0.63 (0.25)	0.28 (1.62)	0 (3.65)	0 (1.05)	0 (0.15)
7	-	-	-	-	-	-	-	-
Test of Homogeneity	47.81 ***		12.73		22.38 ***		21.23 **	-
Score Test for Trend of Odds	30.30 ***	26.57 ***	5.32 *	5.47 *	18.60 ***	18.63 ***	1.66	0.56

* *p* < 0.05; ** *p* < 0.01; *** *p* < 0.001.

## Availability of Data and Material

The data that support the findings of this study are available from Chad Trulson, but restrictions apply to the availability of these data, which were used under license for the current study, and so are not publicly available. Data are however available from the corresponding author upon reasonable request and with permission of Chad Trulson.
